# Community-based surveillance programme evaluation using the platform Nyss implemented by the Somali Red Crescent Society—a mixed methods approach

**DOI:** 10.1186/s13031-024-00578-5

**Published:** 2024-03-06

**Authors:** Julia Jung, Tine Mejdell Larsen, Abdifatah Hussein Beledi, Emi Takahashi, Abdirahman Omer Ahmed, Jenny Reid, Ida Anine Kongelf

**Affiliations:** 1 Norwegian Red Cross, Oslo, Norway; 2 Norwegian Red Cross, now Norad – Norwegian Agency for Development Cooperation, Oslo, Norway; 3 Somali Red Crescent Society (SRCS), Mogadishu, Somalia

**Keywords:** Public health, Community based surveillance, Epidemic diseases, Outbreaks, Epidemic preparedness, Community health, Volunteers, Health risks

## Abstract

**Background:**

Somali Red Crescent Society (SRCS), supported by Norwegian Red Cross, has implemented community-based surveillance (CBS) in Somaliland. This methodology aims to reduce the high risk of epidemics by strengthening early warning and response from and at community level, particularly where there is a weak public health surveillance system. CBS is implemented through SRCS community volunteers, who report signals from the community via SMS to the software platform Nyss. This paper presents key findings from the CBS programme evaluation.

**Methods:**

A retrospective observational mixed-methods approach to evaluate the CBS programme was conducted, using routine CBS data from 2021 for Awdal and Togdheer regions and qualitative interviews with stakeholders’ representatives.

**Results:**

The usefulness of the CBS programme in preventing, detecting, and responding to disease outbreaks was acknowledged by the stakeholders’ representatives. 83% of the signals in Awdal region matched a Community Case Definition (CCD) and were escalated to the Ministry of Health and Development (MoHD)). For Togdheer region, 97% were escalated. Verification of signals by supervisors and escalation to the authorities was done timely.Alert outcome and response action was not well recorded, therefore there is limited evidence on sensitivity. The programme was shown to be simple and can be flexibly adjusted for new diseases and changing CCDs.Stakeholders appreciated being engaged, the good collaboration, their participation throughout the implementation and expressed high acceptance of the programme.

**Conclusion:**

CBS can support early warning and response for a variety of public health risks. Improved documentation for alert outcomes could help to better evaluate the sensitivity of CBS. A participatory approach is vital to achieve successful community volunteer engagement. Software tools, such as the Nyss platform, can be useful to support effective and efficient CBS implementation.

**Supplementary Information:**

The online version contains supplementary material available at 10.1186/s13031-024-00578-5.

## Background

Epidemics begin and end in communities. Often community members are aware of a health threat but the information may be slow to reach health professionals or public health authorities, in turn delaying response. This is particularly true in areas where access to health services is limited and/or there are gaps in the national public health surveillance systems. Community Based Surveillance (CBS) is the systematic detection and reporting of events of public health significance or of cases of a specific disease, within a community, by community members [1]. It facilitates rapid detection and response to outbreaks [2–7].

Previous studies show that CBS can lead to earlier response and confinement of disease outbreaks [6–10]. However, data demonstrating effectiveness of CBS remains limited [11]. Examples of community health workers (CHWs) implementing CBS programmes in hard-to-reach settings, including in armed conflict, have been documented [7, 11–13]. The Red Cross Red Crescent (RCRC) Movement has over 15 million volunteers worldwide, who are members of their local communities [14]. They are linguistically and culturally aligned with the population, and their physical presence within communities make them a strong asset for the implementation of preparedness activities, including CBS, in remote populations where barriers to accessing health exist [6, 8–11].

Somalia, situated in the Horn of Africa, is facing continued armed conflict, population displacement, climate-related shocks leading to recurrent and worsening droughts and famine, and a lack of national funding that have resulted in a deteriorating humanitarian situation in the country [15, 16]. Multiple barriers to accessing healthcare exist. According to the World Health Organization (WHO), the life expectancy in 2019 was 56.5 years while under-five mortality rate, defined as the probability of dying by the age of 5 per 1000 live births, was 111.80 [17, 18]. The main causes of death include lower respiratory infections, neonatal conditions, diarrhoeal diseases, tuberculosis, and measles, which highlight the burden of infectious diseases including vaccine preventable diseases [17].

In Somaliland in particular, there is a high risk of infectious disease outbreaks as well due to the increasing risk of climate change events, poor living and hygiene conditions, limited access to health care, gaps in the national public health surveillance and sub-optimal healthcare seeking behaviour [15, 19]. Disease outbreaks are frequently reported including cholera, measles, chikungunya, scarlet fever, meningococcal meningitis, typhoid, pneumonia and more recently, COVID-19 [20]. All these factors indicate that populations in Somaliland may benefit from CBS to improve early detection and response to infectious disease outbreaks.

Following a large-scale cholera outbreak in Togdheer region in 2017, where there was suboptimal public health surveillance and slow response [21–23], a CBS pilot programme was implemented by SRCS with support of the Norwegian Red Cross in 2018. The programme then expanded to Awdal region in 2020. Currently, CBS is implemented by SRCS in six areas in Somaliland, following needs assessments and upon request of the Ministry of Health and Development (MoHD).

## Programme background

Prior to establishing the CBS programme, a needs and feasibility assessment was conducted, including consultation with communities, local authorities, and relevant partners. The decision for the implementation was then taken based on (i) the historical background that the locations were prone to epidemics; (ii) no other partner had previously or currently implemented similar activities; and (iii) it was possible for SRCS to implement CBS (see Annex 1 for standard CBS assessment guideline and decision-making table for implementation). An implementation strategy was then developed in collaboration with the communities involved and the national and regional MoHD. Principles of CBS implementation described by McGowan et al. [24] were followed, including ensuring a participatory approach with community engagement, local ownership, strong supervision and training and adaptable community case definitions (CCDs).

All community health volunteers (CHVs) were selected and recruited in close collaboration with the communities themselves. Community leaders proposed candidates based on selection criteria provided by SRCS: trusted and accepted by the community, basic literacy, living in the community for long-term, sex and age balanced, willingness to volunteer, commitment to RCRC principles. Based on the geographical spread and population size of the community, two or more volunteers were selected per village and were active in pairs (preferably male and female). Community volunteers were trained in standardised IFRC training packages, adjusted to the context and project, including e.g., community engagement and accountability; epidemic control; and community health and first aid. Based on this foundation of basic knowledge, they were then trained on CBS over 2–3 days. As the volunteers are not health professionals, the training included simple CCDs using signs and symptoms of selected priority diseases. A global list of CCDs has been developed by RCRC Movement partners with Ministries of Health and Agriculture, National Centres for disease control and prevention (CDC), WHO, and other relevant agencies [25]. The CCDs for CBS can be adjusted to align with national CCDs, if these exist in a country as part of national public health surveillance. Table 1 shows the CBS signals and CCDs for the selected reportable disease events by SRCS and MoHD in Somaliland. The criteria for disease events to be included in CBS were those (i) prone to outbreaks in the selected regions that are highly contagious and have high mortality rates; (ii) newly emerging or rare health threats; and (iii) for which a rapid response was possible by MoHD, in collaboration with SRCS or other partners. Community alert thresholds define the number of verified health events required to send an alert to the MoHD for further investigation or response.

**Table 1:** Health signals and community case definitions included in CBS Somaliland until end 2021

Code	Health signal	Community case definition	Health events of public health priority	Community alert threshold
2	Acute diarrhoeal disease (ADD)	Diarrhoea as 3 or more loose or liquid stools over a period of 24 h	Cholera	5 reports within 7 days in 20 km distance
4	Fever and rash	Fever and rash. Often accompanied by or start with runny nose, tiredness, headache, feeling unwell	Measles, Chicken pox	1 report
9	Fever, cough/difficulty breathing, and tiredness	Combination of 3 or more of the following symptoms: Cough; difficulty breathing; fever; runny nose; tiredness; headache; feeling unwell; sore throat; diarrhoea; loss of smell; loss of taste	Covid-19	1 report
14	Cluster of unusual illnesses or death in people	Cluster of people (3+) suddenly sick or died with the same signs of illness in the same village area, in the past 2 weeks.		1 report

Community based volunteers referred sick people to the closest health facility which in some instances are up to 20 kms or they referred to the SRCS mobile clinic which on average visited the targeted village every two weeks.

*Ad hoc* needs-based and regular refresher training were provided throughout the year to maintain quality and motivation. Volunteers of SRCS in Somaliland were not paid salaries or incentivised. When they travelled for training purposes, they received a per diem and transport compensation.

Joint supervision visits to the communities were conducted with MoHD and SRCS management level (CBS manager) on a quarterly basis. During those visits, volunteers were supported by addressing their questions, training needs, or challenges. Communities were engaged during those visits for their feedback and exchange on suggestions, successes, and challenges.

This article aims to contribute to the limited evidence on CBS effectiveness by reporting on findings from the evaluation of the CBS programme through SRCS. The results can provide learnings for other implementations in other contexts as well.

## Methods

This retrospective observational mixed-methods study evaluated the SRCS CBS programme in Somaliland, using quantitative data and qualitative interviews with different stakeholders’ representatives. CBS was fully implemented in Awdal and Togdheer regions in 2021, including rural and urban communities, with 100 volunteers in Awdal from 23 villages, and 139 from 67 villages in Togdheer. Internally Displaced Populations (IDPs) settlements and nomadic populations were included in the community health activities in both regions.

International standards for evaluating surveillance systems were followed [26].

### Quantitative data and analysis

Descriptive analyses of the quantitative data were conducted, using routine CBS data from Awdal and Togdheer regions from January to December 2021. Data was obtained from the Nyss (Norwegian term meaning ‘*having learned or found out about something’*) software platform, a web-based tool specifically designed for CBS and supported by Norwegian Red Cross [27].

Volunteers sent signals on potential health event risks that occurred in their communities based on established CCDs. Using a simple coded SMS on a (non-smart) mobile phone, volunteers were enabled to send a signal to the Nyss platform, even if they were illiterate, spoke a different language, had poor digital literacy, or limited internet connectivity (see Annex 2 for reporting scheme).

If the volunteers did not detect any health risk of concern and didn’t need to send a signal accordingly, they were required to send a zero report at the end of each week. Volunteers’ phone numbers were registered with GPS coordinates to register the geographic origin of a suspected outbreak and facilitate response. These static coordinates represented a random point within a village rather than the reported case of the volunteers’ household to ensure anonymity.

**Fig. 1:**
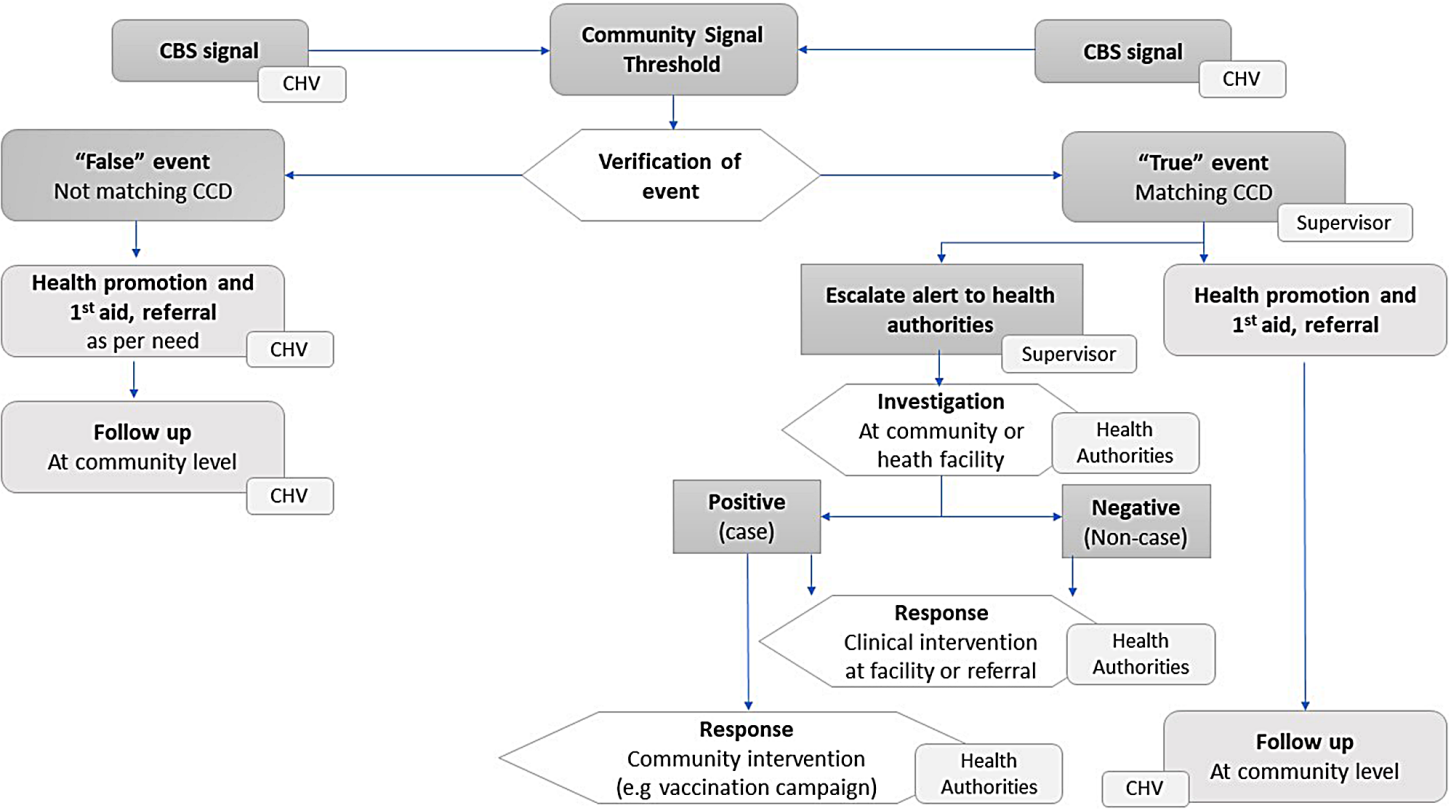
CBS flow diagram

When a signal was sent by a volunteer to Nyss, they received automated feedback via SMS, in their local language, with relevant information on preventive and first aid measures. Once the number of signals from volunteers reached a pre-defined threshold, a notification was triggered to the supervisor to verify whether the signals constituted a public health event of concern. Volunteers’ supervisors are SRCS salaried staff with health-related background and trained in CBS and using the Nyss platform. Supervisors should verify a potential health event within 12 h, which included contacting a volunteer to ensure the signals match the CCD, are not a duplicate or sent incorrectly. The supervisor must assess whether the signal should be dismissed as false or considered as true event to be escalated as an alert to the MoHD for further investigation and response (see Fig. 1 for CBS flow diagram). If the latter, then the MoHD recorded the alert in the national surveillance system. The supervisor was also responsible for supporting and supervising volunteers, including assessing whether a CCD needed to be adjusted for better understanding. The supervisors were supposed to follow up with the volunteer and MoHD on information regarding the outcome of the investigation if available and then close the alert in the system with documentation in the event log on relevant investigation and response information.

Descriptive data analysis was done directly in Nyss, or if further analysis required, in the downloaded Microsoft Excel files available in the platform.

To evaluate the effectiveness of the CBS programme, system attributes from international guidelines [26] and other evaluations [2, 6, 21, 28, 29] were used: usefulness, flexibility, simplicity, data quality, acceptability, sensitivity, and timeliness (see Table 2 for description of the attributes).

**Table 2:** System attributes evaluated

Attribute	Description/rational	Measuring unit/data source	Formula/calculation
Usefulness	CBS can contribute to the prevention and control of adverse health-related events	% of alerts escalated % of alerts responded to Qualitative data from community and MoHD	Alerts escalated or closed (thus previously escalated) that were triggered within the reporting period out of total alerts triggered (also including alerts with the statuses open and dismissed). Escalated alerts that were closed, indicating that a response has happened, whether after referral to the health facility or at community level. Documented response (at least investigation) – by MoH, referral to health facility or national society (e.g., community response by volunteers or mobile clinic). Diagnostic tests or assessments are not the role of SRCS and the volunteers and information is not always available to supervisors and volunteers when documenting the alerts.
Flexibility	Possibility to adjust the programme to include new diseases or case definitions	Key features and events of the programme	NA
Simplicity	System to be as simple as possible to ensure ease of operation	Key features of the programme Proportion of signals matched the CCD in Nyss Qualitative data from volunteers	Kept signals/events of all signals (incl. dismissed/false signals).
Data quality	completeness and validity of the data recorded	% of all reports sent in correct format % of volunteers submitting weekly signals or zero reports	Reports sent in correct format, compared to number of reports sent incorrectly. At least one report per volunteer per week by expected reports from each volunteer.
Acceptability	Willingness of stakeholders to participate in CBS	Qualitative data from the community leaders and MoHD	NA
Sensitivity	The ability of a surveillance or reporting system to detect all true health events	Documented alert outcomes in Nyss: % of alerts that were confirmed after escalation to MoHD	Number of alerts confirmed, compared to all alerts escalated (incl. dismissed by MoHD).
Timeliness	Duration between the actions in CBS	% of CBS events verified within 12 h in Nyss % of alerts responded to with 48 h	Events that were verified by supervisor within 12 h (time between signal was triggered and kept/dismissed) compared to all events verified. Time between escalation and closure of alerts with documented response.

### Qualitative data and analysis

Qualitative semi-structured face-to-face interviews were conducted with different stakeholders’ representatives in December 2021 and January 2022 (see Annex 3 for qualitative interview guideline). Purposeful sampling was used to ensure a range of characteristics of stakeholders’ representatives, including variation in age, sex, experience, and location.

Ten volunteers, seven community leaders, who are respected and for two years elected community members representing the voice of the community (e.g., imams, sheiks, women, and youth leaders) and one key informant from regional MoHD were interviewed. Eight volunteers from different universities, independent from SRCS and partners, were trained on conducting the interviews. Interviews were conducted at community level or at MoHD office in Somali language and were audio recorded, transcribed, and translated to English for analysis. To ensure triangulation, interviews were analysed by using thematic content analysis by one investigator (JJ) and reviewed after by a second (AHB), in consultation with SRCS senior management team and volunteer supervisors. Themes were developed inductively and emerging themes were clustered related to the system attributes.

### Data protection

In Nyss, the SMS reports are only linked to the geographical area the volunteer is registered in. No personal data is collected related to the alerts. Access to personal data of volunteers and RCRC staff in Nyss are restricted to the project and technical staff and are password protected. Only the SRCS team and Norwegian Red Cross technical advisor had access to disaggregated country level data in the Nyss platform.

For the qualitative interviews participants were allocated a study ID. Participants of the interviews gave informed consent in writing to participate in this study. Ethical principles for research were followed.

## Results

### Usefulness

The CBS implementation in Awdal generated 138 signals sent in 2021, with 83% (*n* = 115) alerted to MoHD as verified health events, matching the CCD. With 46% (*n* = 53) most of them were for fever, cough, difficulty breathing and tiredness.

All 115 escalated alerts in Awdal were closed, indicating that some form of response occurred: this might include referral to the health facility for investigation and treatment, investigation at the community level or a public health action (e.g., health education, provision of oral rehydration supplements). However, only 72 (63%) had an outcome documented. Out of those, 61 (85%) had documented “action taken”.

In Togdheer region, 218 signals were generated and 97% (*n* = 213) were escalated after verification. Similarly, to Awdal, most alerted health events were for fever, cough/difficulty breathing, tiredness with 57% (*n* = 122).

All 213 alerts were closed and 136 (64%) had a documented outcome. Out of those, “action taken” was registered for 133 (98%). See Table 3 for detailed distribution.

**Table 3:** Descriptive results of CBS data in Nyss in 2021 in the regions

	Awdal region		Togdheer region	
	n	%	n	%
**Total signals triggered**	138	NA	218	NA
**Events verified within 24 h**	115	83%	213	97%
**Signals dismissed by supervisor, not matching CCD**	23	17%	5	3%
**Alerts escalated**	115	83%	213	97%
*Out of those*				
Fever, cough, difficulty breathing, tiredness	53	46%	122	57%
Fever and rash	31	27%	35	16%
ADD	29	25%	44	21%
Cluster of unusual illnesses and death in people	2	2%	12	6%
**Alerts closed**	115	100%	213	100%
Alerts dismissed by authorities	10	9%	2	1%
Alerts with documented outcome	72	63%	136	64%
*Out of those*				
Action taken	61	85%	133	98%
Documented confirmed positive	/		15	11%
**Total of all reports sent** (signals and zero reporting)	4318		5243	
**Correct format of report**	4144	96%	5210	99%
**Total signals sent**	395		548	
Signals verified	331	84%	507	93%
*Out of those*				
**Verified events—signals matching CCD**	295	89%	502	99%

Qualitative interviews demonstrated that volunteers perceive they can contribute to the prevention and spread of different diseases, as well as improved healthcare services at community level through increasing knowledge on health within their communities.

“*There were several times reported fever and rash and when investigated and found that the reported cases became measles which resulted in response to the community who live in that area and the surrounding communities.*” (Volunteer 9).

Community leaders felt CBS contributed to informed decision making at community level and enabled increased transfer of information to MoHD about challenges in access to health in the communities, where the MoHD have limited access themselves. Community leaders perceived CBS as enabling faster response to disease outbreaks, improving health behaviours and empowering communities to speak up.

“… *there were a lot of people who got sick before the start of Community Based Surveillance, we tried to communicate with the health authority but failed to do so. When Community Based Surveillance started in our community, SRCS trained our volunteers and they report every suspect disease to SRCS and one day, we had children with diarrhoea, just we informed the volunteers, and they shared to SRCS and SRCS notified the health authority. Immediately, SRCS office sent ORS [oral rehydration solution] and Shuban Daweeye [Zinc Tablet] to our community*…” (Community leader 5).

The MoHD key informant recognised the usefulness of CBS to detect disease outbreaks at community level and recognised the rapid CBS reporting system that SRCS is using to alert them.

“*Without CBS, this Measles Outbreak could not have been detected. …it can increase early detection and action to the communities who are already affected by recurrent droughts caused by climate change*” (Government rep).

### Flexibility

When new diseases emerged, SRCS and the MoHD discussed the inclusion of further health signals and CCDs. At the beginning of 2020, a new CCD was included for COVID-19, and volunteers received additional training in detection and reporting. The first patient diagnosed with COVID-19 in Somaliland was detected by SRCS volunteers soon after. CCDs can be adapted, e.g., when volunteers have difficulties in understanding or when knowledge on symptoms or the symptomatic patterns change. This was the case for COVID-19, for which the CCD had to be adjusted in 2021 to align with the WHO definition and based on new knowledge of the disease.

Several changes were made to the Nyss platform during implementation to address gaps or challenges that supervisors and managers had in using the platform, based on user feedback, e.g., inclusion of additional CCDs, additional users, better documentation of outcomes.

### Simplicity

Simple CCDs were utilised in the implementation to ensure that community volunteers could understand and recognise signs and symptoms. Only four CCDs were included at the start of the project and additional ones were added after a period of implementation to ensure volunteers had good understanding.

Simple communication flow was ensured through Nyss features, including simplified reports, automated notifications and analysis of reports. Nyss feedback messages supported the volunteers to respond appropriately before further investigation and response by a healthcare professional.

In Awdal, 395 signals were sent in total (including all signals that reached a threshold and all those that did not but were single signals). Out of all signals, 84% (*n* = 331) were verified by the supervisor and of those, 89% (*n* = 295) were kept as health events, matching the CCD. In Togdheer region, 548 signals were sent in total, out of which 507 were verified, resulting in 99% (*n* = 502) health events (see Table 3).

### Data quality

In Awdal region, a total of 4318 reports (signals and zero reports) were sent from the community volunteers out of which 96% (*n* = 4144) were sent in the correct format (correct reporting code). In Togdheer region, a total of 5243 reports were sent, out of these 99% (*n* = 5210) reports were sent correctly.

**Fig. 2:**
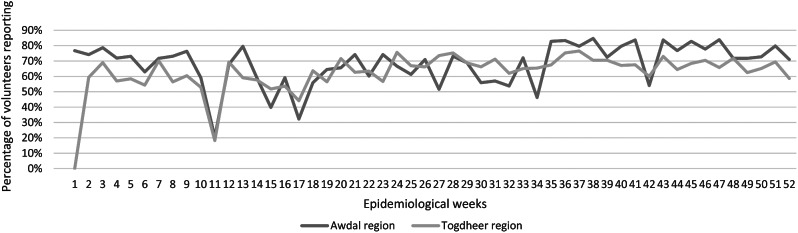
Weekly completeness in both regions in 2021

The percentage of active volunteers submitting weekly reports (signals or zero reports, referred to as ‘weekly completeness’) was at an average of 68% in Awdal region. For Togdheer region, the weekly completeness was at an average of 64%. The lowest percentage of weekly completeness was in the first quarter of the year (see Fig. 2).

### Acceptability

Overall, the CBS implementation in both regions had strong acceptability, and the different stakeholders were willing to participate. There was good collaboration and communication with stakeholders, including healthcare workers, community leaders and community committees. Community leaders were particularly supportive in situations where the volunteers were facing challenges in the community, for example when community members made requests to the volunteers that were outside the scope of their role community leaders were able to support with communication to improve community understanding.

Volunteers faced challenges to physically reach remote communities. As a solution, some volunteers were supported by communities to collect funds towards transport.

“*Yes, we have collaboration with community, the volunteers and we work as a team, the community leaders in our village support us to conduct our activities as per plan and the health workers work with us well, they respect us, and they accept cases we refer to them.*” (Volunteer 1).

Overall, volunteers felt high satisfaction in the CBS programme and were motivated and proud to serve their communities. They felt respected and appreciated by their communities. The support of a supervisor was also key to the motivation of the volunteers. The support volunteers received from their supervisor and SRCS made them feel part of a team and a valued team member whose concerns were taken seriously.

*Community leaders*

Community leaders generally felt ownership for CBS and perceived it as a collaborative activity with SRCS. Community leaders participated in the selection and recruitment of the community volunteers, actively reported to the volunteers when they identified a potential health risk at the community and supported them with challenges in the community to reach the objective of the programme.

“*First of all, the community volunteers are us, starting with me… As I understood, the Community Based Surveillance is made of us*…” (Community leader 1).

Community leaders expressed a wish to expand the programme through more volunteers and geographic locations.

*Health authorities*

The MoHD appreciated their participation in the CBS implementation, including supervision visits and quarterly meetings. The request for extension of the programme to other locations is an indication of acceptability.

“*I would also suggest extending the program to other districts*…” (Government rep).

### Sensitivity

Significant data gaps existed on escalated alert outcomes, which limits the ability to assess the sensitivity of CBS.

Of the 115 escalated alerts in Awdal, 9% (*n* = 10) were documented as “dismissed”, indicating no disease was diagnosed by healthcare professionals. Only 53% (*n* = 61) of alerts in Awdal region had action taken documented, indicating that there was a disease identified, however, none of the alerts had any detailed documentation in Nyss regarding the respective diagnosis.

Of the 213 escalated in Togdheer region, 1% (*n* = 2) were documented as “dismissed”. Of the 133 alerts with documented action taken, 15 (11%) had a laboratory positive test noted by the supervisor indicating a confirmed disease. However, there was no information on which disease/pathogen was diagnosed/detected, and no documentation was recorded for the other remaining alerts (see Table 3).

### Timeliness

#### Verification

All signals triggered in the system were verified by supervisors. Verification took place for Awdal and Togdheer within 12 h in 75% (*n* = 104) 93% (*n* = 202) respectively, while 85% (*n* = 116) and 97% (*n* = 212) were verified within 24 h.

#### Response

The time between an alert being escalated to MoHD and closed was recorded in Nyss. In Awdal, out of all closed alerts (*n* = 115), 67% (*n* = 77) were closed within 48 h. In Togdheer, out of the 213 closed alerts, 76% (*n* = 161) were closed within 48 h.

## Discussion

The results of this study suggest that CBS is a useful, flexible, and simple methodology for contributing to public health surveillance in hard-to-reach communities where access to primary health care is limited. RCRC Movement CBS programmes involve volunteers who invest approximately two hours a week to carry out routine activities in their communities. Sending CBS reports using the Nyss platform is free of charge. The benefit of implementing CBS through community volunteers is that they have direct access and socio-cultural and political knowledge and understanding of the communities within which they live. In the context of Somaliland there are extremely hard to reach communities where there are many barriers to accessing health services. Involving community volunteers mean that epidemic preparedness and response methodologies, such as CBS, can reach these communities. Data in this study supported that the programme is useful in contributing to prevention, detection, and response to outbreaks.

There was a high level of acceptability in the programme by volunteers, communities and the MoHD. Qualitative interviews highlighted good collaboration between different stakeholders and an appreciation of the participatory approach. This resulted in a sense of ownership. Strong community engagement has been shown to be a key factor also in other CBS implementations [2–7, 12, 24, 30]. An expansion of the programme was requested by the stakeholder representatives, further demonstrating the high acceptance.

The accuracy of reporting was shown to be high and implies the CBS approach being a simple methodology for volunteers to implement by using coded SMS. Simple CCDs were well understood by community volunteers and led to high numbers of events that successfully identified unwell people, being escalated as alerts to MoHD. The amount of incorrect reporting via SMS was low and showed further the quality of the CBS data and the benefits of a simple reporting system. Other CBS projects by RCRC movement partners in African countries have shown similar results of reporting accuracy and the perception as a simple approach [6, 30].

Like other CBS implementations [2, 6, 8, 28], the timeliness of verification and response was shown to be very high, particularly considering poor mobile phone network can be a challenge in Somaliland. In the areas evaluated, Togdheer region had slightly better results. This may be because the CBS programme has been implemented longer, as such volunteers and supervisors have participated in more trainings.

The results demonstrated further that CBS has facilitated rapid response to alerts which has been shown also in other CBS publications [2, 28]. The CBS implementation has been integrated as part of wider community health activities, which means that volunteers apply health promotion and first aid, and in some cases integrated community case management (ICCM). The benefits of integrating CBS as part of wider health programmes has been documented in other literature [6, 28].

The CBS implementation strategy was flexible and easy to adjust for new diseases so that it was relevant for changing context or emerging disease patterns. After the study period a CCD for dengue virus was also added and in 2023 the addition of animal diseases has been planned as part of a One Health approach.

However, much improvement can be made in recording outcomes of alerts, to better understand the sensitivity of CBS implementations in contributing to early warning and response as we did not find robust data to make reliable conclusions related to those attributes. In other studies where data was directly accessible from the health facility on the alert outcomes, those conclusions could have been made but still demonstrated to be challenging [28]. In 2021, there was only one major outbreak confirmed in Somaliland through the health cluster, which was an outbreak of measles detected through the SRCS CBS implementation in Togdheer region. There was rapid involvement and collaboration with the MoHD and a swift response. In addition, volunteers routinely provide health information and promotion, which additionally might have contributed to prevention of outbreaks at community level.

A high number of events were detected during the implementation. However, escalated alerts do not provide information on how many of them were confirmed as a disease after investigation, only that actions were taken based on the alert.

The low rate of weekly reporting by volunteers was likely multifactorial. This may be due to lack of motivation in times when there were no concerns regarding a health signal, mobile phone network challenges and lack of charging options, a lack of payments for volunteers’ activities, religious celebrations or movement of volunteers due to climate or nomadic traditions. Weekly reporting was particularly low in the first quarter of the year, when there may have been misunderstandings amongst new volunteers. Refresher training was conducted midway through the implementation, including refresher and update training for supervisors on the Nyss platform as well as solar power banks were distributed to volunteers that had difficulties charging their phones. Byrne and Nichol have discussed similar findings of CBS implementations by non-incentivised RCRC community volunteers in other countries and have pointed out several challenges and potential solutions to address low weekly reporting [6]. CBS implemented through paid community health workers have shown higher rates [7]. A consideration for future CBS implementations dependent on community volunteers is whether weekly reports could be abandoned but built on emphasising that volunteers would at least send signals in case of potential public health risk as a sustainable solution.

The Nyss platform was an effective tool to provide real-time information on potential health risks from the community and to rapidly alert SRCS and MoHD. It provided supervisors and managers with information to monitor and manage the programme activities. The integration of CBS data into national disease surveillance systems has been seen as useful [21] and ensures that data from CBS contributes towards the national surveillance system. The integration into the District Health Information Software 2 (DHIS2) used in Somaliland by the MoHD, is being planned. The Nyss software platform is also continually developed based on user feedback, learnings from other global CBS implementations and consultation with technical experts. Further alignment with the recently published guidelines for early warning alert and response will be done for the RCRC Movement CBS terminology and approach [31].

**Limitations**

Lack of data, particularly on alert outcomes, meant that the sensitivity as the ability of CBS to detect all true health events could not be reliably evaluated. Outbreaks are not always officially declared due to political sensitivity and thus, conclusions are difficult to establish. SRCS works at the community level in Somaliland but is not directly involved in support for healthcare facility services where sick community members are referred to and therefore do not have access to health facility surveillance data where outcomes of investigations are recorded. Diagnostics required for outbreak confirmation is not the role of SRCS and the community volunteers. In addition, there are more systemic limitations in confirming outbreaks. For example, laboratory capacity in Somaliland is limited, thus it may be difficult to confirm an epidemic.

We were not able to obtain qualitative inputs on all system attributes unfortunately. For future evaluations, more specific questions need to be considered in the interview guideline.

A potential bias in the qualitative data cannot be ruled out due interviewer bias. No probing was done during the qualitative interviews and therefore some statements are unclear. In other cases, a potential issue with translations from English to Somali and from Somali to English may have caused misunderstanding or misinterpretations of statements.

Some interviewees may have provided responses relating to the whole project period, not just the period identified for this study. This is a limitation on interpreting the results of the interviews as there may be different events (e.g., active outbreak, refresher training, etc.) that have taken place throughout the project, which may have influenced responses.

## Conclusions

The evaluation of the CBS programme in Awdal and Togdheer region by SRCS demonstrated that CBS can be a useful, flexible, accurate, user-friendly, and timely methodology for supporting early warning and response in hard-to-reach communities. Further expansion of the SRCS supported programme in Somaliland is now being discussed with the MoDH. The results of this evaluation were presented to the SRCS Senior Management Team and have been disseminated to relevant stakeholders. An action plan has been developed to address the prioritised areas for improvement identified.

While the conclusions are drawn from the experience of implementing CBS in only Awdal and Togdheer regions in Somaliland, the results and lessons learned from this study are very relevant and transferable for other contexts. The main recommendations are:

- Community engagement and ownership, at all steps of implementation and effective coordination and collaboration with the MoDH are essential to a successful CBS programme.
- Simple CCDs that can be understood by community volunteers with no specific health experience or training are essential.
- Future CBS implementations should seek to document alert outcomes, where this information is accessible and in coordination with the MoDH. The importance of documenting alert outcomes should also be emphasised in supervisor training.
- CBS should be part of supporting and strengthening national surveillance and health systems. Integrating CBS as part of wider community health programmes has proved successful. As part of the implementation in Somaliland further integration with ICCM is currently piloted.
- There must be ongoing analysis and evaluation of CBS programmes globally, to further understand how early warning systems, epidemic preparedness and response can be improved, particularly in hard to reach communities where there are significant barriers to accessing health care and where the national surveillance system is weak.

### Electronic supplementary material

Below is the link to the electronic supplementary material.

Supplementary Material 1

Supplementary Material 2

Supplementary Material 3

Supplementary Material 4

Supplementary Material 5

## Data Availability

The quantitative datasets of routine CBS from the Nyss platform and the qualitative data from the interviews with the stakeholders are available anonymously from the corresponding author on reasonable request: julia.jung@redcross.no.

## Abbreviations

ADD: Acute Diarrheal disease
CBS: Community Based Surveillance
CCD: Community Case Definition
CHV: Community Health Volunteer
ICCM: Integrated community case management
IDP: Internally Displaced Population
IFRC: International Federation of Red Cross and Red Crescent Societies
MoHD: Ministry of Health and Development
ORS: Oral rehydration solution
RCRC: Red Cross Red Crescent
SRCS: Somali Red Crescent Society
