# Supplementary material for: Community-based surveillance programme evaluation using the platform Nyss implemented by the Somali Red Crescent Society—a mixed methods approach
Source: Confl Health. 2024 Mar 6;18:20. doi: 10.1186/s13031-024-00578-5 (PMC10919031; doi:10.1186/s13031-024-00578-5)
Supplement: Supplementary file 1 — Supplementary Material 1 [file 13031_2024_578_MOESM1_ESM.docx]

**Annex 1**

# Community Based Surveillance – Standard guideline for assessment

Note*: This tool is a guideline! You can adjust the questions, add on, or take out depending on if you have received the information from other sources already several times. During the desk review, you will find relevant information already (e.g., previous declared outbreaks, health risks in the country and project location, health system structure). Depending on the answers, you may want to probe and ask more questions.*

National level

| **Conversation guide with National Society headquarter** | | |
| --- | --- | --- |
| **Structure and activities, CBS experiences and collaboration with MoH** | | |
| **No.** | **Question** |  |
| 1. | What is the structure of the NS related to health/EPR^[[1]](#footnote-2)^/M&E? Roles and responsibilities? |  |
| 2. | Which health/community health/EPR projects has the NS been involved in? |  |
| 3. | What are the experiences of the NS with CBS? Have activities already been implemented? How can CBS be of added value to the projects? Who was involved or has received CBS trainings previously? |  |
| 4. | What is the structure of the health system in the country? How the surveillance system? How is it functioning from your perspective? |  |
| 5. | Are there any initiatives on Community Health/CBS at government level? Which role is/can the NS play in it? How is the NS involved in those initiatives? What are the challenges, gaps etc.?  *Please share the documents of those if available.* |  |
| 6. | What are the main health risks in the country and at project location level? What are the experiences with disease outbreaks? Could you please explain how the preparedness and response by MoH was looking like for those? |  |
| 7. | How is the collaboration with MoH? Would MoH agree to a potential implementation of CBS by the NS? How do you see a potential use of the CBS platform Nyss by the NS (*if applicable*)? |  |
| 8. | How is the NS envisioning the implementation of CBS into the project(s)? Who will be involved and how? |  |
| 9. |  |  |
| **Conversation guide with Ministry of Health (+ WHO)** | | |
| **Health & surveillance system, epidemic preparedness & response, CBS experiences & willingness** | | |
| 1. | Please explain roughly the health system in the country. How is the surveillance system functioning? Which surveillance tools are used? Roles and responsibilities. Data flow and response. Where are the gaps in the surveillance system (*if any*)? *(Check if there any kind of surveillance system from the community level, if it wasn’t mentioned yet)* |  |
| 2.a | Is CBS part of the national health policy or planned to be? If yes, how is it (supposed to be) implemented? Which reporting tools are used? How is it functioning in terms of early reporting? How does it influence early response? |  |
| 2.b | Are there already other partners in the country implementing CBS with the MoH? Where, how? Please share contacts.^[[2]](#footnote-3)^ |  |
| 3. | Do you have existing Community Case Definitions for the communities to identify health risks and events? Do you have training or other materials available (e.g., indicators)? |  |
| 4. | How is the response mechanism functioning if a health risk is discovered? What happens if investigation confirms an outbreak? Roles and responsibilities. Timeframe for investigation and response interventions *(How quick is investigation and response happening, where are the challenges?).* |  |
| 5. | The NS is assessing if CBS is needed and feasible in their (community health) projects to support MoH in the early detection and reporting of potential infectious diseases at community level. NS would include the national policies/materials in their implementation (if applicable) and link with existing surveillance tools. Would MoH appreciate and agree that the NS would take this activity into their community health programme? Are there any concerns about CBS? |  |
| 6. | … |  |
| **Conversation guide with other actors** *(if relevant) national level and project level!!!* | | |
| **CBS programmes experiences, collaboration with MoH, lessons learned** | | |
| 1. | What are your experiences with CBS programming in the country/location? Where are or have you been implementing? |  |
| 2. | Please explain me more about the strategy that you were/have been using: reporting mechanisms, human resources, what, when, where, who, how many, sustainability, acceptance from community etc. |  |
| 3. | What is your experience with MoH collaboration? How is the response mechanism functioning? |  |
| 4. | What are your lessons learned (other than already mentioned) and what would you recommend to new CBS implementations in the country? |  |
| 5. | *If CBS is implemented by another partner in the same location, discuss on needs/ways to collaborate. E.g., is there a gap RCRC volunteers can fill within CBS? Can we support with detection, (electronic) reporting, etc.* |  |
| 6. | … |  |

District/project level

| **Conversation guide with National Society (branch)** | | |
| --- | --- | --- |
| **Structure and activities** | | |
| **No.** | **Question** | **Response** |
| 1. | What is the structure of the branch? Staff, volunteers - roles and responsibilities? |  |
| 2. | With which (health) projects has the branch been involved or is currently involved? |  |
| 3. | Can you describe the activities and work of the volunteers? (e.g., activities, how much do they work, geographical area, incentives, previous trainings – what and when) |  |
| 4. | How do you monitor the activities? (area, transportation, capacity, data collection, ITT, M&E plan - ask to see documents) |  |
| 5. | How do the volunteers report on their activities? (data collection tools, frequency, reporting lines - ask to look at reports) |  |
| **Health risks** | | |
| 1. | What do the volunteers do if someone is sick? Does the branch get to know about it? |  |
| 2. | What are the main public health risks in your coverage area? |  |
| 2. | How do you respond to the public health risks and needs in your area? |  |
| 3. | How do you prepare for and respond to disease outbreaks? Do you share information with other branches/districts? |  |
| 4. | When was the last time an outbreak was declared in your district/project location? Can you please describe what happened before and after? How did you get to know about it? How was it responded to? (incl. within which timeframe) |  |
|  |  |  |
| **CBS and communities** | | |
| 1. | What do you know about CBS? |  |
|  | Do you believe there is a need for CBS in your area? Why? For what? |  |
| 2. | How do you see CBS being integrated into your existing activities? |  |
| 3. | What capacity do you think is necessary and do you believe the branch has it? (data management, response, collaboration, volunteer support, data sharing) |  |
| 4. | What is the mobile network coverage in your area? Can volunteers read and write? How good is access to electricity? |  |
| 5. | Do you believe the volunteers would be interested/receptive to CBS? |  |
| 6. | From your perspective, how will the communities accept CBS? Which challenges do you foresee? |  |
|  |  |  |
| **Other actors in location** | | |
| 1. | Please describe access to health care in the locations: distance, services, quality, needs/gaps etc. How you work with them. |  |
| 2. | Which other organisations are working in your project locations? What are they doing? At community level? How do you collaborate with them? |  |
| 3. | Which health programmes does MoH provide at community level? How do you collaborate with them? |  |
| 4. | If other/similar programmes are implemented, where do you see possibilities of collaboration, gaps/needs, how we can complement? |  |
| 5. |  |  |
| **Conversation guide with Ministry of Health (district level)** | | |
| 1. | What are the prioritized health risks in your district? |  |
|  | Do you have existing Community Case Definitions for the communities to identify health risks and events? |  |
| 2. | Can you describe reporting through the surveillance system? E.g., EWARS, IDSR? Use of DHIS2 or other digital HIS? Ho is the data collection fucntioning? (At which levels, tools, etc.) Any documents or training materials available? Data/information flow and response? Data protection?  Timeliness of investigation/response; availability and processing of lab results. |  |
| 3. | Are there any areas/communities which are not in the catchment of health facilities or other health services? What is done for these areas to ensure they are covered with health services. |  |
| 4. | Can you describe how the data collected are used? (analyzed, shared, actions, reporting lines) (Any reports to see?) |  |
| 1. **Outbreak response** | | |
| 1. | How and when is a disease outbreak declared? |  |
| 2. | When was the last time an outbreak was declared in your district? Can you please describe what happened before and after, incl. how did you get to know about it? |  |
| 3. | How was it responded to? (incl. within which timeframe) |  |
| 4. | Which actors are active in the community with which activities? |  |
| 5. |  |  |
|  |  |  |
| 1. **CBS** | | |
| 1. | Do CHWs or other community members participate in surveillance? If yes, how? |  |
| 2. | Are there any health surveillance activities done at the community level? |  |
| 3. | Do you believe there is a gap in the surveillance system in terms of getting information from community level? In which communities/areas? Why? |  |
| 4. | Do you think CBS could have an added value and feed into your existing surveillance system? Why? How? (incl. which health risks/events) |  |
| 5. | What is the capacity of the district to manage and respond to alerts from the community? Data management? Case verification and community-based investigation? Intervention? Communication and coordination? |  |
|  |  |  |
| **Conversation guide with National Society volunteers – group interview** | | |
| 1. **Working as a volunteer** | | |
| 1. | Can you talk about the health-related activities you do in your community? Please describe how you carry out these activities and with what frequency. |  |
| 2. | How were you trained to do these activities? What trainings have you received (incl. from other organisations)? |  |
| 3. | What are the geographical areas you cover? How do you move around? |  |
| 4. | How are you working with the communities? What are the challenges? |  |
| 5. |  |  |
| 1. **Communication and reporting** | | |
| 1. | How do you communicate with the branch and the National Society? Who is your main point of contact? Do you have regular communication? |  |
| 2. | How do you report on your activities? Do you report anything else? |  |
| 3. | What do you do if you hear of someone in your community being sick?  Do you report to someone? To whom and what? What happens then? |  |
| 3. | Do you have/use a mobile phone? How do you charge it? How reliable is the network coverage in your area? How good is access to electricity for charging your phone? |  |
| 4. | How do you reach your supervisor, how do you reach a health care worker? |  |
|  |  |  |
| 1. **Health and health risks** | | |
| 1. | How close or far is the nearest health clinic from your community? |  |
| 2. | Are there any community health workers (CHW) or village health teams (VHT) in the community? What kind of activities are they doing? Are you a community health worker? |  |
| 3. | Can you talk about what happens if someone in the community gets sick? Do people go to the health facility? (access, cost) Are there traditional healers? Access to pharmacies? |  |
| 4. | If something serious was happening in the community (death, disease, disaster): Who in the community would be the first to know? How would YOU find out? What do you do if something serious is happening in the community? Has this already happened to some of you? What did you do? |  |
|  |  |  |
| **D. Conversation guide with health facility staff and/or CHW** | | |
| 1. | What is the area you cover? Population size and catchment area if known? |  |
| 2. | Do you think everyone in this area can easily get to you? (access, cost, trust) Are there some populations you know that do not come to the clinic if they are sick? What do they do? |  |
| 1. **Health risks and surveillance** | | |
| 1. | What are the health problems you see in this area? Do you have any reports, register? Can we see what you have? Are there any seasonal differences? |  |
| 2. | If someone comes to you with a communicable disease, is this reported in any surveillance system? |  |
| 3. | *If, yes to question 2*: Can you describe reporting through the surveillance system? How do you collect data? Where? Do you use digital or paper registers? Do you get data from other areas about cases or outbreaks? How is the data transmitted to the MoH? What happens after the reports? |  |
| 4. | What happens if you report, for example, a case of measles or other communicable disease? Is there an investigation? By whom? How fast? Can you give an example of a previous case? How do you participate? What was the response? |  |
| 5. | What happens if someone dies in the community? |  |
| 6. | Do you believe there is adequate and timely detection of potential outbreaks? If yes, why? If not, why not? What are the gaps? What do you think should be done? |  |
| 1. **CBS** | | |
| 1. | Do the health facility and CHWs participate in surveillance? If yes, how?  Do you get information from the community on sick people? If yes, how and from whom? |  |
| 2. | If someone is sick in the community, do you have the capacity to go there and verify? (number of staff, outreach activities)/ are you going there to verify? |  |
| 3. | Do Red Cross Red Crescent volunteers often refer to you? How do you work with them? |  |
| 4. | Have you heard of CBS? What does it mean for you? |  |
| 5. | Do you think that the Red Cross Red Crescent could play a role in CBS? If yes, what kind of role? If no, why not? What are the barriers? |  |
| 6. | Do you think CBS could be useful/have an added value in your area? How can you picture it adding value? |  |
| **Conversation guide with community members** | | |
| 1. **Structure** *(can be done as observation as well and might be known. This is not CBS specific questions but Community health and might have been assessed already)* | | |
| 1. | How is your community structured: distances within community (household to household, to public places like markets etc.)? Distance to next community? Distance to health facility? Who is/are the community leader(s)? Mobile network coverage, electricity? |  |
| 2. | Do you have health committees? Community health workers? Red Cross Red Crescent volunteers? How are you working with them? |  |
| 1. **Health risks** | | |
| 1. | Can you discuss what health risks you see in your community? |  |
| 2. | Can you talk about what happens if someone in the community gets sick or dies? Do people go to the health facility? (access, cost) Do you work with the health facilities? Traditional healers? Do you report to anyone (whom)? |  |
| 3. | Can you talk about how the community would respond to a disease outbreak? |  |
| 4. | Have you ever had a disease outbreak in your community? What happened? How was it responded to? |  |
| 5. | What do you think would be the most important support you could give to your community to prevent disease outbreaks or during a disease outbreak? |  |
| 1. **Partners** | | |
| 1. | How do you work with Red Cross, Red Crescent? Are there volunteers present in the community? If so, do you know what they do? |  |
| 2. | Are there other organizations doing activities here? Which ones? What are their activities? How do they work with you? |  |

# Community Based Surveillance – Decision making table

| **Conclusions/way forward, e.g.,** | **Yes/ feasible** | **Possible, but hard** | **No/ unfeasible** |
| --- | --- | --- | --- |
| **1. Need or relevance of CBS**  - Is there a gap in surveillance at the community or primary health care level?  - Would CBS be a useful tool to fill this gap? |  |  |  |
| **2.** **Suitability:**  - If there is a need, can the NS fill it and is it the appropriate partner to fill that need in the current context?  - If a national level CBS programme is being implemented, can the NS join these efforts? |  |  |  |
| **3. Feasibility for NS to implement a CBS project**  - Based on its capacity – or what size of project would be feasible to implement, and what type of support would be needed.  - Are there staff who could be involved in the CBS project. Additional training needs for staff and volunteers?  - Would additional support be required?  - Are funding mechanisms identified to support CBS? |  |  |  |
| **4. Openness of country health authorities to collaborating on CBS activities**  - Is MoH+other actors open to RCRC playing a role in CBS?  - Can a partnership with the MoH be established for investigation of cases and response?  - Does MoH or other actors have capacity to respond to CBS. |  |  |  |
| **5. Potential modality, structure, health risks and sites for a RCRC CBS project**  - Does/can the RCRC work in the locations identified as key sites to implement CBS?  - Have considerations been made regarding the modality of data collection, feasibility, and data flow |  |  |  |

1. Emergency Preparedness and Response [↑](#footnote-ref-2)
2. In case there are, link up with them and explore how they are doing, lessons learned, collaboration with MoH on early response etc. [↑](#footnote-ref-3)
