# Supplementary material for: Community-based surveillance programme evaluation using the platform Nyss implemented by the Somali Red Crescent Society—a mixed methods approach
Source: Confl Health. 2024 Mar 6;18:20. doi: 10.1186/s13031-024-00578-5 (PMC10919031; doi:10.1186/s13031-024-00578-5)
Supplement: Supplementary file 2 — Supplementary Material 2 [file 13031_2024_578_MOESM2_ESM.docx]

**Annex 2: SMS reporting scheme**

| 1. Digit:  **Health risk/event #** | 2. Digit:  **Sex #** | 3. Digit:  **Age** |  |
| --- | --- | --- | --- |
| *[insert the right health risk and number],* e.g.  1= Acute Diarrheal disease Diarrhea  or  4 = Fever and rash | 1 = Male  2 = Female | 1 = 0 – 4 years old  2 = 5 or more years old | *Examples:*  A man with many frequent stools last night  SMS: **2#1#2**  A girl of 3 years with fever and rash all over the body  SMS: **4#2#1**  A 22years old woman who experiences fever, cough and difficulties in breathing and feels very tired.  SMS: **9#2#2** |
| 14= Cluster of unusual illnesses or death in people | NA | NA | *Example:*  Seven children with puffed out cheeks with high fever in one community during the past five days  SMS: **14** |
| 99 = Zero report [no health risk/event detected but active] | NA | NA | The volunteer has not found any health risk in her community during this week.  SMS: **99** |
