# Supplementary material for: Community-based surveillance programme evaluation using the platform Nyss implemented by the Somali Red Crescent Society—a mixed methods approach
Source: Confl Health. 2024 Mar 6;18:20. doi: 10.1186/s13031-024-00578-5 (PMC10919031; doi:10.1186/s13031-024-00578-5)
Supplement: Supplementary file 3 — Supplementary Material 3 [file 13031_2024_578_MOESM3_ESM.docx]

**Annex 2: Comprehensive qualitative interview tools**

# SRCS Community Based Surveillance Evaluation

# Participant Information Sheet – December 2021

## Introduction

We would like to invite you to take part in an evaluation assessment. Joining this assessment is entirely up to you. Before you decide, you need to understand why the evaluation is being done and what it would involve. I will go through this information sheet with you, and answer any questions you may have. Ask questions if anything is not clear or you would like more information.

## What is the purpose of the study?

SRCS is conducting this assessment to evaluate the Community Based Surveillance (CBS) project, which aims to improve the detection and response to Acute Diarrheoal diseases (e.g., Cholera), to Measles, and other infectious diseases.

## Why have I been asked to take part?

You have been invited because you were/are involved in the CBS set up and/or implementation. Your views are important to understand if the CBS is functioning effectively to reduce diseases in your community.

## Do I have to take part?

No. It is up to you to decide if to take part. If you don’t want to take part, that’s ok. You will still continue to be involved in the CBS. We will discuss the study together and give you a copy of this information sheet. If you agree to take part, we will then ask you to sign a consent form.

## What will happen if I take part?

You will be asked questions about what you know about CBS and how you think it could be improved. You will be interviewed by two independent data collectors, one to ask questions and the other to record your responses. The interview should take no more than 30 minutes.

## What are the possible benefits?

The information we get from this study will help improve the CBS and improve detection and response to some diseases in your community.

## What if something goes wrong?

If you have a concern about any aspect of this assessment, you should ask to speak to the interviewers who will do their best to answer your questions. Further, SRCS can be contacted: Abdifatah Hussein Beledi, Phone: +252 634848485, Email: [beledi05@gmail.com](mailto:beledi05@gmail.com); Kaltuun Hussein Daher, Telephone: +252 (0) 63 4410092, Email: [kaltuun2002@yahoo.com](mailto:kaltuun2002@yahoo.com); Hassen Abdi Jama, Telephone: +252 (0) 63 4417229, Email: [haj2010@hotmail.com](mailto:haj2010@hotmail.com). They can be contacted both in English and Somali, and also by telephone.

Julia Jung, Email: [Julia.Jung@redcross.no](mailto:Julia.Jung@redcross.no) for questions about the study design itself, can be contacted by email. She can be contacted in English.

## Can I change my mind about taking part?

Yes. You can withdraw from the study at any time. You do not need to give a reason to withdraw from the study.

## What will happen to information collected about me?

All information collected will be kept private. Only the study staff will have access to the information. Your data will be stored securely.

## What will happen to the results of this study?

The study results will be for internal use to improve the CBS activities and potentially published in a report and research journals so that other public health practitioners can learn from them. Your personal information will not be included in the study report and there is no way that you can be identified from it.

## Who has checked this study?

All research involving human participants is looked at by an independent group of people, called a Research Ethics Committee, to protect your interests. This study has been reviewed and given favourable opinion by Dr. Mohamed Abdi Hergeye, the director general, Ministry of Health Development, Republic of Somaliland. Ref: MOHD/DG:2/125/2018.

## Further information and contact details

Thank you for taking time to read this information leaflet. If you think you will take part in the study please read and sign the consent form.

If you would like any further information, please contact Hassen Abdi Jama, who can answer any questions you may have about the study.

Contact details:

Hassen Abdi Jama, Telephone: +252 (0) 63 4417229.

**SRCS Community Based Surveillance Evaluation**

**Informed Consent Sheet**

**Name of Persons responsible for project:**

Abdifatah Hussein Beledi, Phone: +252 634848485, Email: [beledi05@gmail.com](mailto:beledi05@gmail.com)

Kaltuun Hussein Daher, Telephone: +252 (0) 63 4410092, Email: [kaltuun2002@yahoo.com](mailto:kaltuun2002@yahoo.com);

Hassen Abdi Jama, Telephone: +252 (0) 63 4417229, Email: [haj2010@hotmail.com](mailto:haj2010@hotmail.com)

Julia Jung, telephone: +254 793 466 282, Email: [Julia.Jung@redcross.no](mailto:Julia.Jung@redcross.no);

| **Statement** | **Please initial or thumbprint* each box** |
| --- | --- |
| I confirm that I have read and understood the information sheet dated December 2021 for the above named study. I have had the opportunity to consider the information, ask questions and have these answered satisfactorily. |  |
| I understand that my consent is voluntary and that I am free to withdraw this consent at any time without giving any reason and without my involvement in the CBS project being affected. |  |
| I understand that data from me may be shared, and that I will not be identifiable from this information |  |
| I agree to taking part in the above named study. |  |

Printed name of participant Signature of participant Date

|  |  |  |
| --- | --- | --- |

(or thumbprint/mark if unable to sign)

Printed name of person obtaining consent Signature of person obtaining consent Date

|  |  |  |
| --- | --- | --- |

(or thumbprint/mark if unable to sign)

| **SRCS-NorCross Evaluation of Functionality & Effectiveness of Community Based Surveillance in Somaliland** | |
| --- | --- |
|  |  |
| **Community Stakeholders Interview Guide (Community Elders & Community Health Committee members)** | |
|  |  |
|  | **Study ID for Community Stakeholder:** |
|  | **Region: Togdheer Awdal District: Buhoodle Borama Zeila Lughaya** |
|  | **CBS Village:** |
|  | **Position/designation:** |
|  | **Sex:** |
|  | **Interviewer:** |
|  | **Date:** |
|  |  |
|  | **Question** |
| 1 | What do you know about Community Based Surveillance in your community? *Probe: Understanding of CBS purpose, experiences, local volunteer/s and volunteer/s roles and activities* |
|  |  |
| 2 | 1. How are you involved in CBS? 2. How would you like to be involved? |
| 3 | 1. Who are the CBS volunteers in your community? 2. How do you work together with the volunteers? 3. How do you work together when there are health issues in the community? |
| 4 | 1. How does the community perceive the activities the volunteers are doing in CBS? 2. How is the rest of the community engaged in Community Based Surveillance? 3. How could the community be engaged more or differently? |
| 5 | If you neighbor’s child has fever and rash on her body. What would you do? |
| 6 | Do you think Community Based Surveillance has benefitted the community or will benefit the community?  If so, how? *Probe: Detection of diseases/outbreaks, faster and more effective response to outbreaks, more resources, improved health of community.*  If not, why?  *Probe*: The aim of CBS is early detection and warning of health authorities on infectious diseases like Measles or Cholera and others, to respond faster to avoid huge outbreaks. From your experience with CBS, how has CBS reached this goal in your community?  Please give an example. |
| 7 | Have you seen any challenges or concerns with Community Based Surveillance? *Probe: Not enough information or feedback, low community acceptance or support, lack of response, lack of feedback back from CBS team/volunteers*  How can they be overcome? |
|  |  |
| 8 | What can you and your community do to sustain the positive change that CBS activities supported by SRCS have brought to your community? |
|  |  |
| 9 | Do you have suggestions for how Community Based Surveillance could be improved going forward? *Probe: Expand diseases/illnesses, more feedback on how CBS information used, better communication with CBS team, use CBS data to get better health services in community.* |
| 10 | Any other comments regarding community based surveillance? |

| **SRCS-NorCross Process Evaluation of Functionality & Effectiveness in Community Based Surveillance in Somaliland** | |
| --- | --- |
|  |  |
| **Government Stakeholders Interview Guide** | |
|  |  |
|  | **Study ID for Government Stakeholder:** |
|  | **Location:** |
|  | **Designation: Institution:** |
|  | **Sex:** |
|  | **Interviewer:** |
|  | **Date:** |
|  |  |
|  | **Question** |
| 1 | What do you know about the Community Based Surveillance project by SRCS in your region? *Probe: Understanding of CBS purpose, structure, function, local volunteer/s and volunteer/s roles and activities.* |
|  |  |
| 2 | a) How were and are you involved in the Community Based Surveillance project?  b) How would you like to be involved? *Probe: Inception and/or planning meetings, selection of volunteers, problem-solving implementation of CBS.* |
| 3 | 1. How do you see the added value of CBS in the communities where SRCS/ICRC has implemented the project?   *Probe: Detection of diseases/outbreaks, faster and more effective response to outbreaks, improved health of community*   1. Where do you see the added value of CBS for the MoH?   *Probe: How has CBS contributed to early detection and early response to infectious diseases in those communities? How has CBS informed your actions from the MoH?*  *Probe*: The aim of CBS is early detection and warning of health authorities on infectious diseases like Measles or Cholera and others, to respond faster to avoid huge outbreaks. From your experience with CBS, how has CBS reached this goal in your communities?  Please give an example.   1. How else could CBS add value to the early detection and warning on epidemic diseases in your communities? |
|  |  |
| 4 | Where do you see the challenges, concerns, or gaps with the CBS programme?  How could they be overcome? |
|  |  |
| 5 | 1. What Community Based Surveillance information do you receive? 2. How has it informed your actions? *Probe: Type of information received, frequency, types of action, any outbreaks declared, impact on actions (earlier)* |
|  |  |
| 6 | Do you have suggestions for how Community Based Surveillance could be improved going forward? *Probe: Expand diseases/illnesses, more feedback on how CBS information used, better communication with CBS team, use CBS data to get better health services in community.* |
| 7 | Any other comments on community-based surveillance? |

| **SRCS-NorCross Process Evaluation of Functionality & Effectiveness in Community Based Surveillance in Somaliland** | | |
| --- | --- | --- |
|  |  |  |
| **Community Volunteer Assessment** | |  |
|  |  |  |
|  | **Study ID for Community Volunteer:** |  |
|  | **Age:** |  |
|  | **Sex: Female Male** |  |
|  | **Region: Togdheer Awdal**  **District: Ainabo Burao Odweine Buhoodle**  **Borama Zeila Lughaya** |  |
|  | **CBS Village:** |  |
|  | **Interviewer:** |  |
|  | **Date:** |  |
|  |  |  |
|  | **Question** | **Response** |
|  |  |  |
| 1 | When did you start reporting as a Community Based Surveillance Volunteer? *Single response only* | ----------------------(MM/YYYY) |
| 2 | What are your roles and responsibilities in this project as CBS volunteers? *Multiple responses Prompt: "Anything else?"* | - providing health education in the community  - reporting on sick people in the community  - providing first aid, referring sick people to the health facility  - helping the health care workers with the response  - Other:  - I do not know |
|  | *Case scenarios* |  |
| 3 | While you do your health promotion activities in the communities, you are hearing about the following cases: | |
| 3.1 | A girl of 58 months with high fever and rash on the whole body.   1. What are you reporting for this situation? 2. Which other actions are you doing after reporting? *Multiple responses Prompt: "Anything else?"* | 1. Fever and rash;   4#2#1  Other answer  I do not report  I do not know  Ask if child is vaccinated for measles  Advise on isolation of the child to avoid the spread  Advise on hygiene measures  Refer child to the health facility  Call supervisor for support/advise  Other:…  I do not know |
| 3.2 | A 22-years old man with rice water stools.   1. What are you reporting for this situation? 2. Which other actions are you doing after reporting? *Multiple responses Prompt: "Anything else?"* | 1. ADD, 2#1#2, suspected Cholera   Other answer  I do not report  I do not know   1. Provide or show ORS preparation   Advise on hygiene measures  If dehydrated, refer to health facility  Call supervisor for advice  Alert family on reporting similar symptoms to me  I will pay attention on the neighborhood/village if there are other sick people with the same symptoms and will report  Other  I do not know |
| 3.3 | A 55-years old woman returns from a visit of her family in Hargeisa. Two days after being back home, she develops headache and tiredness, fever, and loss of taste.   1. What are you reporting for this situation? 2. Which other actions are you doing after reporting? *Multiple responses Prompt: "Anything else?"* | 1. 9#2#2, suspected Covid-19   Other answer  I do not report  I do not know   1. Advise on isolation   Advise on wearing a mask  Advise on hygiene measures  Refer to the health facility if symptoms are severe  Advise to get tested for Covid 19  Provide awareness on signs and symptoms for other family,  Provide awareness on vaccination if applicable,  Other:  I do not know |
| 3.4 | A child under 5 has experienced bloody stools for 2 days.   1. What are you reporting for this situation? 2. Which other actions are you doing after reporting? *Multiple responses Prompt: "Anything else?"* | 1. I do not report   Other answer:  I do not know   1. Advise on hygiene   Refer to the health facility if symptoms are severe  Other:….  I do not know |
| 4 | What are the signs and symptoms of suspected Covid-19 patients? | Combination of 3 or more of the following symptoms:  ● Cough  ● difficulty breathing  ● fever  ● runny nose  ● tiredness  ● headache  ● feeling unwell  ● sore throat  ● diarrhoea  ● loss of smell  ● loss of taste  Only two symptoms mentioned  Different symptoms mentioned  Other:  I do not know: |
| 5 | How do you find out about someone being sick in your community? *Multiple responses Prompt: "Anything else?"* | Relative/s of suspected case = 1 Friends = 2 Community elders = 3 Community Health Committee = 4 Local authority = 5 Other (please specify) = 6 I have not sent any SMS to report a health risk =7  I have not come across any sick person in my community which  No response = 88 Don't know = 99 |
|  |  |  |

**Thanks for your participation and the valuable input you gave!!**

**Qualitative questions ONLY FOR a SELECTED number VOLUNTEERS (please ask the study responsible):**

**I have a few extra questions for you. Please express whatever comes into you mind.**

| Q1 | How are you collaborating with…   1. …the communities and community leaders? 2. …with the health care workers of the mobile and static clinics 3. …with your supervisor |  |
| --- | --- | --- |
| Q2 | 1. What are the challenges in your activities as volunteer? 2. How can they be overcome? 3. Which support do you wish to get from your supervisor? |  |

| Q3 | | What motivates you to be a volunteer for CBS? *Probe: "Is it important to feel valued/ to see impact of your work/ have training opportunities/ visible recognition of efforts/ incentives?"* |
| --- | --- | --- |
| Q4 | | What do you know about the impact and results of your work in CBS? |
| Q5 | | Where do you see the benefits of Community Based Surveillance for your community? |
| Q6 | | What are your suggestions to improve the CBS project? |
|  | |  |
| Q7 | What motivates you to be a volunteer for CBS? Probe: "Is it important to feel valued/ to see impact of your work/ have training opportunities/ visible recognition of efforts/ incentives?" | |
| Q8 | What do you know about the impact and results of your work in CBS? | |
| Q9 | Where do you see the benefits of Community Based Surveillance for your community? | |
| Q10 | What are your suggestions to improve the CBS project? | |
