# Supplementary material for: Community-based surveillance programme evaluation using the platform Nyss implemented by the Somali Red Crescent Society—a mixed methods approach
Source: Confl Health. 2024 Mar 6;18:20. doi: 10.1186/s13031-024-00578-5 (PMC10919031; doi:10.1186/s13031-024-00578-5)
Supplement: Supplementary file 4 — Supplementary Material 4 [file 13031_2024_578_MOESM4_ESM.docx]

Annex 5: additional quotes from stakeholders during qualitative interviews per system attribute

Usefulness

*“…what we do is to prevent the disease to spread in our community or to know exactly that there is health problem in the community to tell red crescent or the government. The trainings we are provided and the work we are conducting in our community had an impact and good results and improves the quality of healthcare”.* (Volunteer 3)

*“About the immunization, previously you would see that the people were against immunizing their children and the father and mother had different opinions about it, now it is different. I could say all these achievements were the result of information sharing and awareness rising provided by red crescent and the trainings they provided members of our community.”* (Community leader 1)

*“I believe CBS has added value to the existing health system, it can increase early detection and action to the communities who are already affected by recurrent droughts caused by climate change...the health and hygiene promotion initiatives of SRCS volunteers improved the capacity of their respective communities for prevention, early detection and facilitated health seeking behaviour…The way SRCS is capturing information is an advanced system through which volunteers are sending reports immediately when they receive a case. This will help us to be informed if an outbreak is coming up in that area, so we closely watch out for reports they share with us.”* (Government representative)

Acceptability

“*The community respect [motivates us] because if the community do not respect you, that is challenging and it will lead you demoralized…, [the community] provides us the necessary support we need all the time..*.” (Volunteer 2)

*“I am confident, and I know what I am telling my community and any question raised from my information shared, I have the answer. The continuous contact from my supervisor and the regular supervision provided me motivation because red crescent they did not train only one time and they did not say go for work, but they come to us, sit with us and we discuss to talk about the community, the challenges we have and if they can be solved immediately, take the necessary steps… It motivates me to see that we are not neglected but we are caring as much as they can.”* (Volunteer 4)

*“…if this project with the red crescent stops, our activities will not stop”* (Community leader 1).

*“At the beginning of the Community Based Surveillance, we participated in the planning…we were appointed to select the members who will be working on the community-based surveillance.”* (Community leader 1)

*“But rarely, unwise people ask for to provide them non-food items or cash, think that community volunteers are paid or was given medical items to distribute to the community and sometimes, say if you do not have something to provide as please leave us and this is not the right thing and when the inform us the next day, we go with the volunteer to the same family who rejected to listen them and provide further explanation about their role and responsibility and their works as volunteerism and this helped a lot.”* (Community leader 4)

*“The number of volunteers trained for our community is not sufficient and they cannot cover all” (Community leader 4)*
