# Supplementary material for: Community-based surveillance programme evaluation using the platform Nyss implemented by the Somali Red Crescent Society—a mixed methods approach
Source: Confl Health. 2024 Mar 6;18:20. doi: 10.1186/s13031-024-00578-5 (PMC10919031; doi:10.1186/s13031-024-00578-5)
Supplement: Supplementary file 5 [file 13031_2024_578_MOESM5_ESM.docx]

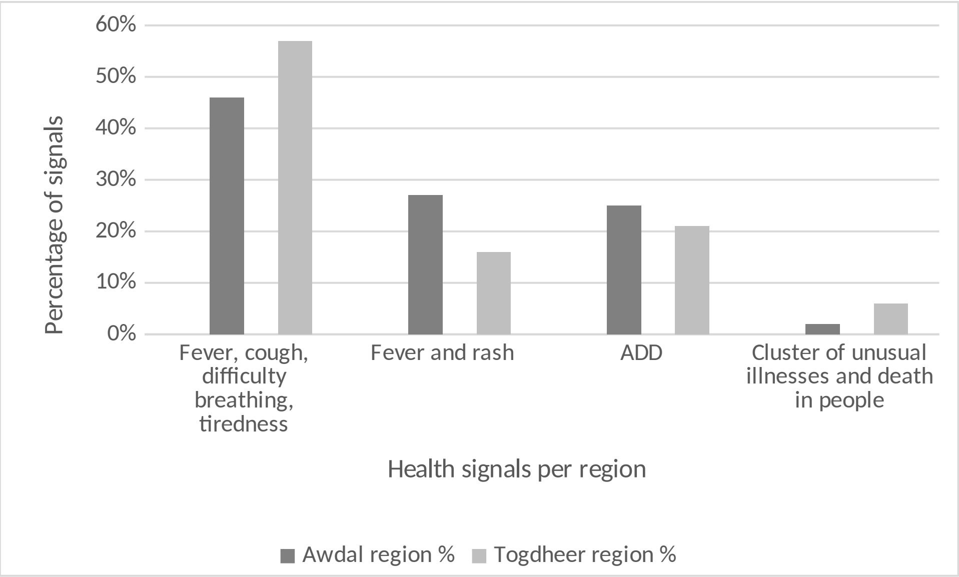
60%
50%
40%
30%
20%
10%
0%
Percentage of signals
Fever, cough, difficulty breathing, tiredness
Fever and rash
ADD
Cluster of unusual illnesses and death in people
Health signals per region
Awdal region %
Togdheer region %
